# Right Paraduodenal Hernia and Review of CT Signs to Diagnosis: First Case Report in Viet Nam

**DOI:** 10.70352/scrj.cr.25-0345

**Published:** 2025-09-26

**Authors:** Phu Thanh Lam Nguyen, Nhat Ba Minh Nguyen, Nghia Ngoc Do, Duy Hoai Nhat Nguyen, Trung Quoc Pham, Duc Van Nguyen, Phuc Hung Nguyen, Tri Thanh Anh Le

**Affiliations:** 1 Department of General Surgery, Binh Dan Hospital, Ho Chi Minh, Viet Nam; 2 Department of General Surgery, Pham Ngoc Thach University of Medicine, Ho Chi Minh, Viet Nam

**Keywords:** acute abdomen, paraduodenal hernia, bowel malrotation, bowel obstruction, CT, Ladd procedure

## Abstract

**INTRODUCTION:**

Paraduodenal hernia (PDH), a rare cause of acute abdominal pain in the emergency department, is difficult to diagnose and can cause serious issues by mimicking common conditions.

**CASE PRESENTATION:**

We present a 49-year-old male with a right PDH causing a 2-day history of nausea, bowel obstruction, and periumbilical/right abdominal pain. CT revealed bowel obstruction and a right PDH, confirmed intraoperatively. A Ladd procedure was performed, and the patient recovered well.

**CONCLUSIONS:**

This case highlights specific CT signs that were instrumental in diagnosing this rare disease, ultimately guiding a successful treatment strategy for the patient.

## Abbreviations

PDH
: paraduodenal hernia
SMA
: superior mesenteric artery
SMV
: superior mesenteric vein

## INTRODUCTION

PDHs, also called mesocolic hernias, are a specific type of internal hernia, where an abdominal organ abnormally protrudes through a peritoneal or mesenteric defect. This uncommon condition arises from incomplete rotation of the midgut during fetal development. PDH, however, is the most common type of internal hernia, constituting 53% of internal hernia cases; however, they cause only 0.2%–0.9% of all cases of intestinal obstruction.^1,2)^ Clinically and surgically, PDH is present in 2 significant forms: left-sided, involving small intestine prolapse through Landzert’s fossa, and right-sided, where bowel herniation occurs via Waldeyer’s fossa.^3,4)^ Right PDHs are rarer than their left-sided counterparts, making up less than 25% of all PDH cases. This represents a particularly rare occurrence, as fewer than 50 cases have been recorded in PubMed since 1970, and it is the first such report from Viet Nam.^4,5)^ The symptoms of internal abdominal hernias vary depending on whether the herniated bowel loops can spontaneously return to their normal position. This can range from indistinct upper abdominal pain when the hernia reduces on its own, to severe, cramping pain around the navel when the bowel becomes trapped and its blood supply is compromised.^2)^ The diverse range of symptoms associated with PDH can mimic other causes of acute abdominal pain seen in the emergency department. This overlap makes PDH a diagnostically challenging condition for clinicians. In these situations, a strong suspicion is necessary, and a CT scan can help establish an accurate diagnosis, especially in emergency cases.^3,6)^ We present the case of a complicated PDH in a patient who presented with acute intestinal obstruction and signs of bowel gangrene on a CT scan. A Ladd procedure was performed. This report adheres to the SCARE criteria.^7)^

## CASE PRESENTATION

A 49-year-old man presented to the emergency department with a 2-day history of continuous, diffuse abdominal pain, most intense around and to the right of his navel, which severely restricted his activity. He also reported multiple episodes of bilious vomiting and complete constipation. He had no prior abdominal surgeries, injuries, or known medical conditions, but mentioned experiencing occasional pain after eating. Physical examination revealed tenderness in the right abdomen and periumbilical region, and rebound tenderness. Auscultation revealed normal bowel sounds and slight abdominal distention was observed.

Laboratory analysis showed only a high white blood cell count of 21.41 K/uL. Initial X-rays and ultrasounds of the abdomen were inconclusive, leading to an urgent pre- and post-contrast CT scan of the abdomen and pelvis, reconstructed in multiple views without oral contrast.

**Fig. 1:**
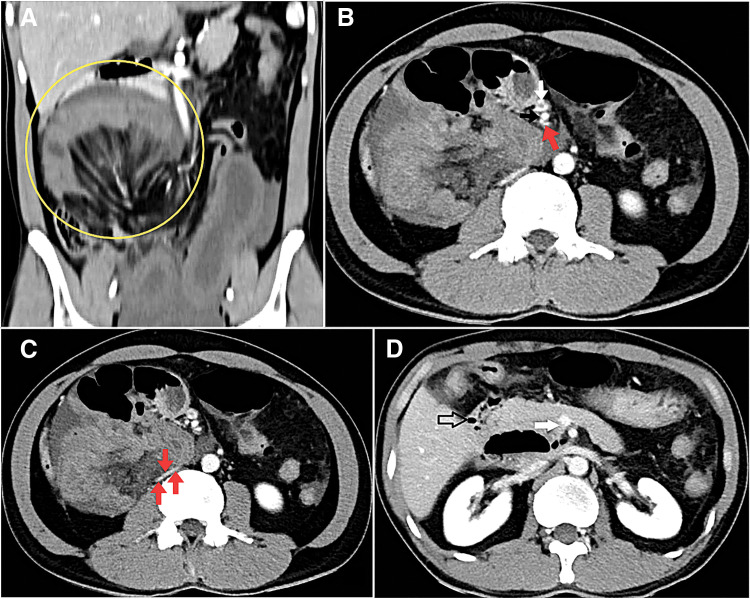
CT scan on admission. (**A**) On coronal images, the small intestine is displaced to an abnormal position near the right abdominal wall (yellow circle), with the right colon absent from its normal anatomical location. (**B**) Axial images showed the jejunal artery and vein branches (red arrow) wrapped from the front, around the SMA (black arrow), to its posterior right (SMV–white arrow). (**C**) The inferior vena cava (red arrows) was compressed by a hernia sac. (**D**) The 3rd part of the duodenum (open arrow) did not run to the left side, and the SMV (white arrow) is on the right side of the SMA. SMA, superior mesenteric artery; SMV, superior mesenteric vein

CT scan revealed an abnormal position of the transverse colon and right colic flexure, with small bowel loops situated above and to their right (**Fig. 1A**). Additionally, the jejunal artery and vein branches originate from the front, wrap around the SMA, and then extend toward the back and to the right of the SMA (**Fig. 1B**). The hernia sac, which encloses small intestine loops, also compresses the inferior vena cava (**Fig. 1C**). The 3rd portion of the duodenum was not visualized in its typical anatomical location. Moreover, the SMV is in its usual position to the right of the SMA, contrary to what would be expected with malrotation (**Fig. 1D**).

The patient initially received non-operative management with intravenous fluids and antibiotics before immediate transfer to the operating room for surgical intervention.

**Fig. 2:**
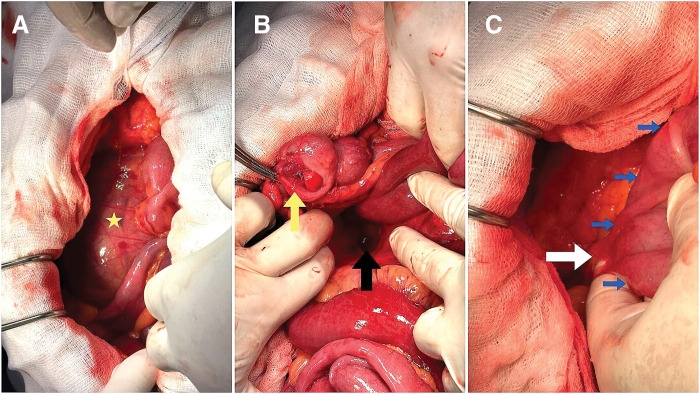
(**A**) Upon entering the abdomen, a hernia sac (star) was found to the right of the ascending colon, containing dilated small bowel loops. (**B**) Dissection revealed the small bowel had herniated through a defect adjacent (black arrow) to the right side of the duodenum. The herniated small bowel was free. The appendix was held by a kelly for better exposure (yellow arrow). (**C**) After that, further exploration of the right abdomen revealed that the cecum (blue arrows) flexure was adhered to the right abdominal wall by a Ladd’s band (white arrow). The Ladd’s band was subsequently divided.

**Fig. 3:**
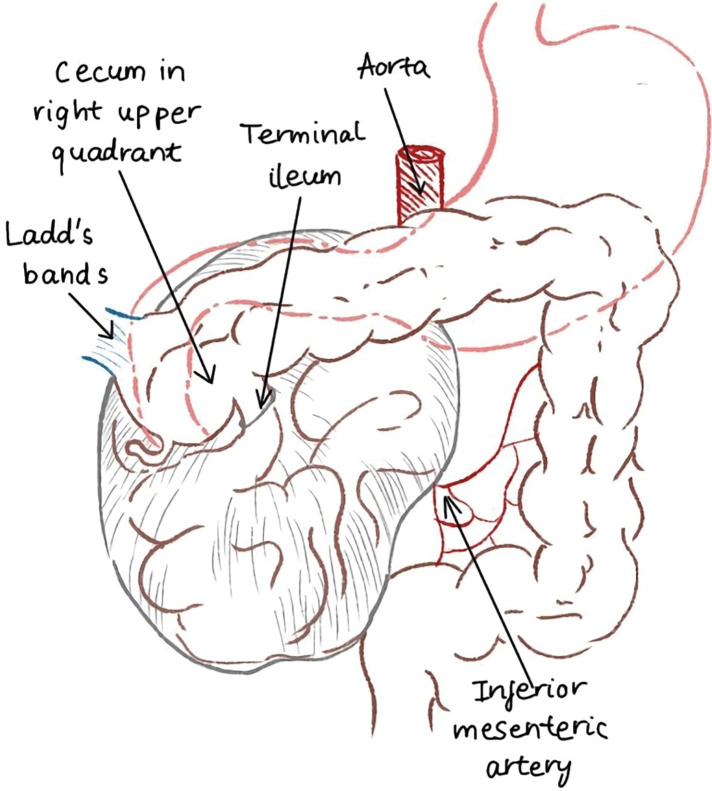
The intraoperative illustration shows the relative positions of the colon, Ladd’s band, and the hernia sac.

Following the induction of general anesthesia, a restricted midline incision was made below the umbilicus, the linea alba was incised, and access to the peritoneal cavity was established, followed by the creation of pneumeopritoneum using an open Hasson technique. However, the presence of distended small bowel loops along with hemorrhagic and turbid fluid within the peritoneal cavity necessitated the conversion of the laparoscopic approach to an open laparotomy. The hernial sac, containing loops of the small intestine, was located on the right side of the ligament of Treitz (**Fig. 2**). Evidently, a congenital defect in the vicinity of Treitz’s ligament constituted the opening of the internal hernia, a finding that substantiated the diagnosis of a right PDH. The small intestine that was trapped within the hernia sac was freed. After being covered with warm gauze, it regained its healthy pink color. A Ladd procedure was performed, involving the untwisting of the intestines and the division of Ladd’s band. An appendectomy was also carried out. In **Fig. 3**, the diagram of the whole colon and small intestine, including the hernia sac, in operative findings shows the degree of rotational abnormality, the small intestine entering the hernia orifice, and the relative positions of the colon, Ladd’s band, and hernia sac. The patient’s postoperative recovery was uncomplicated, and he was discharged after 5 days.

**Fig. 4:**
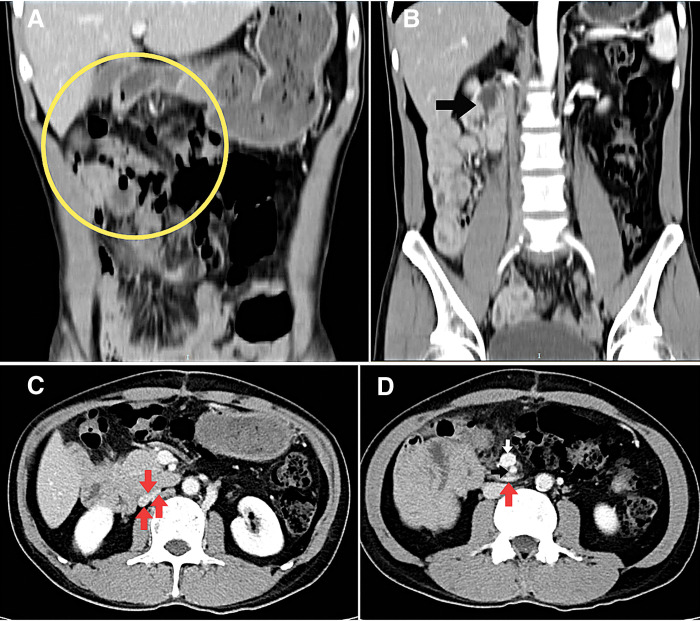
(**A**) No signs of mesenteric congestion or fat stranding were observed (yellow circle). (**B**) The duodenum’s 3rd part and duodenojejunal flexure (black arrow) remained on the right, not crossing the midline. (**C**) The inferior vena cava (red arrows) was no longer compressed. (**D**) There were no changes observed in the position of the jejunal artery and vein branches (red arrow) (SMA–black arrow, SMV–white arrow). SMA, superior mesenteric artery; SMV, superior mesenteric vein

One month postoperatively, a follow-up CT scan was conducted using the identical scanning protocol as the initial examination. There was no evidence of mesenteric congestion or fat stranding (**Fig. 4A**). The 3rd portion of the duodenum and the duodenojejunal flexure were still located on the right side of the abdomen and did not cross the midline (**Fig. 4B**). The inferior vena cava was no longer experiencing compression (**Fig. 4C**). The positions of the jejunal artery and vein branches were unchanged between the preoperative and postoperative CT scan (**Fig. 4D**). The patient reported no new symptoms during the follow-up period.

## DISCUSSION

PDHs are a type of internal hernia where an abdominal organ protrudes through a defect in the peritoneum or mesentery. Although they are the most common type of internal hernia, they account for only 0.2%–0.9% of all intestinal obstruction cases.^2)^ PDHs are more common in males, at a 2:1 ratio, and can be diagnosed at any age. There are 2 clinically significant types: the more common left-sided hernia through Landzert’s fossa and the rarer right-sided hernia through Waldeyer’s fossa, which constitutes less than 25% of all PDH cases.^3,6)^ This particular case is a rare occurrence, with fewer than 50 right PDH cases reported in PubMed since 1970, and this being the 1st such report from Viet Nam.^4,5)^

PDHs typically form during embryonic development due to abnormal midgut rotation and fixation, making them congenital anomalies often linked to intestinal malrotation. PDHs develop when the midgut’s normal 3-stage rotation process during fetal development is disrupted, specifically during the 2nd stage. This disruption leads to both small bowel malrotation and the formation of a PDH. At that point, the midgut re-enters the abdominal cavity from the yolk sac. The small bowel is then positioned on the right side of the abdomen, having already completed a 90-degree counterclockwise rotation. Normally, the small bowel completes another 180-degree counterclockwise rotation, settling behind and to the left of the superior mesenteric artery. If this rotation does not occur, a segment of the small bowel stays on the right side of the superior mesenteric artery, becoming enclosed in a hernial sac behind the colonic mesentery, thus forming a right PDH.^4)^ Therefore, the SMA and SMV serve as the defining landmarks for the right PDH, running along the anteromedial border of the fossa.^3)^

Right PDH presents with a variety of nonspecific symptoms, ranging from chronic to acute abdominal pain, often accompanied by nausea and vomiting, and occasionally fever. Patients might also report a history of recurring bowel obstructions since childhood, weight loss, or, in some cases, may not experience any symptoms at all.^2)^ In the emergency department, this wide range of symptoms makes PDH a diagnostically challenging condition as it can mimic other causes of acute abdominal pain. This diagnostic difficulty is a critical aspect of PDH management. For instance, differential diagnoses for acute abdominal pain could include appendicitis, cholecystitis, pancreatitis, or other forms of intestinal obstruction.^2,3)^ In this case, the patient’s symptoms and physical findings, including diffuse abdominal pain, vomiting, and complete constipation, were consistent with bowel obstruction. Since initial abdominal X-rays and ultrasounds did not identify the cause of the pain, a CT scan was utilized.

A CT scan is instrumental in establishing an accurate diagnosis, especially in emergency situations. Previous literature has identified 5 typical CT signs for diagnosing a right PDH^3,4,6,8)^:

- As a sac-like cluster of dilated small bowel loops in the right upper abdomen’s Waldeyer’s fossa, with the duodenum’s 3rd part superior and the mesentery root anterior;
- The displacement of the SMA: the jejunal artery and vein branches move to the posterior right of the SMA, and the SMV at the anteromedial edge of the fossa;
- Surrounding structures are compressed by a hernia sac, such as the ureter, inferior vena cava, and right psoas muscle;
- The 3rd duodenum will not cross the left;
- The SMV will be on the left of the SMA if malrotation is present.

In our patient’s case, the CT scan findings corresponded to several of these diagnostic signs. The scan revealed small bowel loops located above and to the right of the transverse colon and right colic flexure. The jejunal artery and vein branches originated from the front, wrapped around the SMA, and extended toward the back and right of the SMA. A hernia sac containing small intestine loops compresses the inferior vena cava. The 3rd portion of the duodenum was not visualized in its typical anatomical location.

However, one finding on our patient’s CT scan deviated from the expected signs of malrotation often associated with PDH. The SMV was in its usual anatomical position to the right of the SMA. This is contrary to what is typically expected with malrotation, where the SMV is positioned to the left of the SMA. This specific observation highlights a key aspect of diagnostic complexity, as not all classic signs of malrotation may be present in every case of PDH.^3,8)^

Our finding that the SMV and SMA retained their normal anatomical relationship has also been noted in a 2019 study, which states that SMA–SMV positional changes are not always present if there is no malrotation. Despite this difference, both our case and the previous study showed a key shared finding: the jejunal artery and vein branches were displaced to the posterior right of the SMA.^3,8)^

Moreover, CT scan also presents obstruction complications that manifest as dilated loops, air-fluid levels, mesenteric congestion, and fat stranding and helps exclude other causes or identifies coexisting conditions.^3,4,6)^

During surgery for a right PDH, the small intestine is moved back to its normal position after the 1st stage of intestinal rotation. The defect is then either closed or the opening of the hernia is made larger. However, directly opening the hernia sac should be avoided because the superior mesenteric vessels are very close to the opening, and doing so could damage the intestine’s blood supply or cause significant bleeding.^4,5)^ In our case, surgeons performed a Ladd procedure, which included untwisting the intestines and cutting Ladd’s band.^5,9)^ An appendectomy was also completed. An appendectomy is typically performed during this procedure for 2 key reasons: the appendix’s unusual placement in the left upper quadrant can complicate the diagnosis of appendicitis, and there is a risk of damage to the appendiceal artery when dissecting the Ladd’s band.^10)^

## CONCLUSIONS

Right PDHs are very rare, yet can range from incidental findings to life-threatening bowel obstructions. An immediate CT scan is crucial for diagnosis in both acute and chronic cases. Early and accurate diagnosis, especially through careful CT scan review, is vital, as surgery is the only way to prevent severe intestinal complications.
